# Effects of fun games on respiratory function and compliance in children with pneumonia: a randomized controlled trial

**DOI:** 10.3389/fpubh.2026.1824969

**Published:** 2026-09-07

**Authors:** Ying Chen, Xin He, Jin Gong, Qin Wang, Ting Chen, Yanyun Zhuang, Liqin Ye

**Affiliations:** Department of Pediatrics, Shenzhen People's Hospital (The First Affiliated Hospital, Southern University of Science and Technology; The Second Clinical Medical College, Jinan University), Shenzhen, China

**Keywords:** children, fun games, pneumonia, rehabilitation compliance, respiratory function

## Abstract

**Background:**

Community-acquired pneumonia poses a significant threat to children's health. Traditional respiratory rehabilitation often suffers from poor adherence due to children's limited cognitive abilities. This study aimed to evaluate the effects of a fun game-based intervention on respiratory function, exercise capacity, and rehabilitation compliance in children with pneumonia.

**Methods:**

A single-center, two-arm, parallel-group randomized controlled trial was conducted. A total of 170 children recovering from pneumonia and meeting the inclusion criteria were recruited from a tertiary hospital in Shenzhen and randomly allocated to either an intervention group or a control group. Ultimately, 154 participants (77 per group) completed the study. The control group received conventional respiratory training and health education. The intervention group participated in an 8-week fun game-based program, including respiratory training games (e.g., “Blow Out the Little Candle”), physical activity games (e.g., “Rhythmic Breathing Run”), along with interactive feedback mechanisms and personalized adjustments. Pulmonary function (FEV1/FVC ratio), exercise capacity (6-min walk distance, 6MWD), and rehabilitation compliance (Frankl and Houpt scale scores) were measured in both groups before and after the intervention.

**Results:**

No statistically significant differences were found in baseline characteristics or outcome measures between the two groups before the intervention (*P* > 0.05). After the 8-week intervention, the intervention group showed significantly greater improvement in pulmonary function (FEV1/FVC: 84.69 ± 2.34 vs. 74.43 ± 2.27, *P* < 0.001) and exercise capacity (6MWD: 593.63 ± 19.91 m vs. 556.35 ± 30.48 m, *P* < 0.001) compared to the control group. Regarding compliance, post-intervention Frankl scores (3.87 ± 0.34 vs. 3.10 ± 0.60) and Houpt scores (2.91 ± 0.86 vs. 3.19 ± 0.78) indicated significantly better cooperation willingness and treatment completion in the intervention group (*P* < 0.05).

**Conclusion:**

The fun game-based intervention significantly improves pulmonary function and exercise capacity, while effectively enhancing compliance and completion rates in the rehabilitation of children with pneumonia. This approach is economical, feasible, and effective, offering a novel strategy for clinical nursing and rehabilitation practice in pediatric pneumonia.

**Clinical trial registration:**

https://www.chictr.org.cn/showproj.html?proj=269965

## Introduction

Recent findings in ***The Lancet*** identify childhood pneumonia as a global public health threat (1). Pneumonia remains the leading infectious cause of child mortality worldwide. Children are particularly vulnerable due to their immature immune systems and developing respiratory tracts (2). According to World Health Organization (WHO) data from 2019 (2), pneumonia resulted in over 700,000 child deaths, with a disproportionate burden in developing countries. In China, childhood pneumonia incidence is approximately 21 million cases, the world's second highest (3). The disease often follows a prolonged and recurrent course, with 7% to 13% of cases progressing to severe, life-threatening illness requiring hospitalization, This severely compromises children's growth, development, and quality of life while placing a substantial burden on their families. Although advances have been made in diagnosis, treatment, and prevention, persistent challenges include constrained medical resources and antibiotic resistance (3).

Chest physiotherapy is a crucial adjunctive measure in managing most respiratory diseases, with pulmonary rehabilitation forming a fundamental component (4). Pulmonary rehabilitation is the core of respiratory disease management in children (5). However, the American Thoracic Society (ATS) has indicated that pediatric pulmonary rehabilitation differs from adult programs and must be tailored to children's age and cognitive abilities (6). Effective pediatric pulmonary rehabilitation requires individualized adjustments based on the child's cognitive capacity, whereas the traditional clinician-led model may struggle to meet this requirement. For children with limited cognitive and comprehension abilities, this approach often fails to stimulate active participation, leading to poor adherence and suboptimal rehabilitation outcomes.

Game is an innate aspect of childhood. By incorporating gaming elements into engaging activities, game-based interventions allow children to complete respiratory rehabilitation tasks in a relaxed and enjoyable atmosphere. Compared with standard care, constructing a therapeutic environment that emphasizes key concepts of self-determination theory and delivers interventions that are novel, engaging, and scaffolded can enhance children's engagement (7). Although the fun-based game-guided model has shown success in managing other conditions (8, 9). Its application in pediatric pneumonia respiratory rehabilitation remains exploratory. This study therefore investigates how this model promotes compliance and efficacy in the respiratory rehabilitation of children recovering from pneumonia, offering new perspectives for improving outcomes in this population.

## Methods

### Study design

This 8-week, two-arm, parallel-group randomized controlled trial was preceded by a planning phase to finalize the fun games intervention protocol (Table 1).

**Table 1:** Fun games intervention protocol.

Phase	Training content	Format	Time	Frequency	Supplementary materials
Intervention initiation	1. Etiological triggers of pneumonia 2. Significance of respiratory training 3. Design logic of therapeutic games 4. Demonstration of a game operation	PowerPoint presentation (Guardian+child)	60 min	1. Time (before intervention)	-
Intervention phrase	1. Daily: explain the knowledge from that day's game after the session 2. Weekly: review the week and hold a Q&A session	-	1.5–10 min; 2. 20 min	1. Once daily (QD) 2. Once weekly (QW)	Health handbook (game illustrations, problem record sheet, goal sheet)
After invention	1. Knowledge questionnaire test 2. Satisfaction interview and collection of suggestions for improvement	Questionnaire + interview (guardian)	1. Questionnaire: 20 min 2. Interview: 15 min	Once every 4 weeks, for a total of 2 sessions	Questionnaire

### Randomization and allocation concealment

This study used block randomization with a fixed block size of 4. The allocation sequence was generated using a web-based randomization service (www.randomization.com). To ensure allocation concealment, the sequence was placed in sequentially numbered, opaque, sealed envelopes. A staff member who was not involved in participant recruitment or outcome collection opened the envelopes only after each participant had completed all baseline assessments and was formally enrolled.

### Blinding

Due to the nature of the intervention, it was not feasible to blind the interventionists or the participants to group assignment. However, we made every effort to minimize potential bias: (1) outcome assessors were kept blind to group allocation throughout the study; (2) all intervention procedures were delivered using standardized scripts and protocols; and (3) data analysis was performed by a researcher who was unaware of group codes until the final analysis was completed.

### Study subjects

Children in the recovery phase after pneumonia were enrolled between May 1, 2025, and September 30, 2025. Eligible participants had non-severe pneumonia and had achieved clinical stability before receiving the rehabilitation intervention. Children with severe pneumonia requiring intensive respiratory support or mechanical ventilation, or those with severe complications, were excluded. Inclusion criteria were: (1) a clinical diagnosis of pneumonia, confirmed by laboratory and imaging findings and classified (10); (2) age between 3 and 12 years; (3) clinical stability, defined as normal body temperature for ≥3 days, substantial resolution of symptoms (e.g., cough, tachypnea), and absence of radiographic progression, as determined by a physician with >10 years of clinical experience; and (4) provision of written informed consent by a parent or legal guardian.

Exclusion criteria were: (1) presence of severe complications (e.g., empyema, respiratory failure necessitating mechanical ventilation); (2) concomitant major systemic diseases (e.g., congenital heart disease, immunodeficiency disorders); (3) cognitive impairments precluding comprehension of game instructions (e.g., intellectual disability); (4) any other condition severely limiting participation in the play intervention (e.g., severe musculoskeletal disorders, active infectious diseases); and (5) concurrent enrollment in another clinical study.

### Sample size

A two-sided test with a significance level (α) of 0.05 and a power (1-β) of 0.80 was used to ensure statistical power. Based on preliminary pilot data, a medium effect size (Cohen's d = 0.5) was anticipated for the improvement in nursing compliance and treatment outcomes with the game-guided model. G^*^Power software calculated a required sample size of 68 participants per group. To account for potential attrition, this number was increased by 20%, yielding a final total sample of 170 participants to ensure the reliability of the findings.

### Data collection

All primary analyses were performed according to the per-protocol (PP) principle, including only participants who completed the intervention without major protocol deviations. Data were collected at multiple time points throughout the study. Baseline assessments were conducted 1 day prior to the intervention. Forced vital capacity (FVC) and forced expiratory volume in one second (FEV1) were measured by uniformly trained technicians using pediatric spirometers. The 6-min walk test (6MWT) was administered by designated nurses in an indoor corridor. Baseline demographic and clinical characteristics, including age, gender, disease duration, and initial treatment adherence, were recorded by the same trained nurses through parental interviews. Final outcome data were collected upon completion of the 8-week intervention, repeating all baseline measurements and documenting total adherence and completion rates for the study period. To ensure data integrity, research assistants performed weekly cross-checks of all collected data.

### Ethical review

All participants consented to data anonymization. The study was approved by the Ethics Committee of Shenzhen People's Hospital (Approval No. LL-KY-2025074-01) and registered with the Chinese Clinical Trial Registry (Registration No.: ChiCTR2500101623).

### Intervention methods

In our clinical setting, pulmonary rehabilitation is routinely incorporated into the management of children recovering from pneumonia, particularly for those with residual respiratory symptoms or impaired exercise capacity after clinical stabilization. However, standardized pulmonary rehabilitation protocols for pediatric pneumonia remain insufficiently established. Previous studies have demonstrated that respiratory rehabilitation interventions lasting several weeks can improve respiratory function, exercise capacity, and treatment adherence in children with respiratory diseases (4, 5). Therefore, considering the time required for children to adapt to rehabilitation activities and achieve measurable functional improvement, an 8-week intervention period was selected in this study. The intervention was a structured, game-guided respiratory rehabilitation program delivered over 8 weeks. Its core component was daily respiratory rehabilitation through age-appropriate games designed to train breathing control, improve ventilation, and promote respiratory coordination during physical activity. The games were selected from a standardized intervention manual and were matched to the child's developmental level and rehabilitation goals.

The intervention was delivered by rehabilitation therapists and responsible nurses. Rehabilitation therapists were responsible for selecting appropriate games, demonstrating the correct breathing and movement techniques, adjusting the difficulty level when necessary, and ensuring that the activities met the intended rehabilitation goals. Responsible nurses assisted with daily implementation, provided brief health education before and after the sessions, monitored safety and child cooperation, and completed fidelity checklists after each session.

Caregivers supported the intervention during home practice. Their role was to help the child complete the assigned games, encourage cooperation, record session completion and duration, and report any difficulties encountered during home training. Caregivers were not allowed to independently modify the intervention content or intensity; any necessary adjustment was discussed with the rehabilitation therapist or responsible nurse.

To improve reproducibility and intervention fidelity, all interventionists followed a standardized operating procedure manual and completed training before implementation. Each session was checked using a fidelity checklist, with 85% set as the minimum acceptable score. The research team also conducted weekly supervision, including review of intervention progress and random observation of selected sessions either in person or by video recording. Adherence was assessed using daily attendance records and caregiver-completed home training logs. Health education was used as a supporting component rather than the core intervention. On the first day of intervention, caregivers received a 60-min group session explaining the rationale for respiratory rehabilitation and the use of game-guided training. During the 8-week intervention, nurses provided brief education before and after daily sessions, and a 20-min weekly reinforcement session was held to review home practice, address caregiver-reported difficulties, and reinforce correct training methods. A Health Handbook was provided to caregivers, including illustrated game instructions, common rehabilitation questions, and weekly goal-setting tables (Table 2).

**Table 2:** Intervention protocol for games.

Intervention methods	Core dimensions	Specific content
Respiratory training games	Implementation Frequency	Once daily, 6 consecutive days per week, with 1 day of rest
	Duration per session	10–15 min
	Specific content	1. “Blow Out the Little Candle”: blow out 3–5 virtual candles to earn reward stickers 2. “Digital Windmill Challenge”: blow the digital windmill as instructed; earn a sticker after 3 consecutive correct attempts
	Implementation process	1. Prepare the items 5 min in advance 2. During the intervention, observe breathing movements and correct errors (e.g., avoid shrugging shoulders, guide abdominal breathing) 3. After the session, provide immediate feedback (verbal encouragement) and record task completion (number of levels completed, achievement rate)
Physical activity games	Implementation frequency	Once daily, with a 2–3 h interval between respiratory training and physical activity (respiratory in the AM, physical activity in the PM), 6 consecutive days per week, with 1 day of rest
	Duration per session	15–20 min
	Specific content	1. “Animal Breathing Exercises”: imitate animals stretching, etc., coordinated with “inhale—hold breath for 2 seconds—exhale” 2. “Balloon Relay Race”: parents and children form a team to pass a balloon using their abdomens 3. “Simulated Flight Game”: spread arms to imitate an airplane, performing ascent and descent actions as instructed 4. “Rhythmic Clapping Breathing Exercise”: clap hands while exhaling, raise hands while inhaling, following the music 5. “Breathing Rhythm Run”: “Run 3 steps—Inhale, Run 3 steps - Exhale” (5 min of running + 1 min of rest, for a total of 3 rounds)
	Implementation process	1. Check the safety of the venue in advance and explain the rules and safety precautions 2. During the intervention, monitor heart rate and breathing (if heart rate exceeds the upper limit by 10 beats/minute or if shortness of breath occurs, pause immediately) 3. After the session, guide a 5-min cool-down (slow walking, deep breathing)
Interactive feedback mechanism	Implementation frequency	Synchronized with respiratory/physical activity games (immediate feedback after each session); Periodic Feedback
	Specific content	1. Immediate Feedback: award points based on performance (10 points per task for respiratory training, 15 points for meeting physical activity goals). Points can be exchanged for rewards (Ages 3–6: 100 points for stickers/toys; 150 points for comic books/stationery; Ages 10–12: 200 points for reading materials/wristbands), accompanied by verbal encouragement 2. Periodic Feedback: On Sundays, review the week's participation (points, completion rate) with the family, analyze progress and areas for improvement, adjust games according to the child's preferences, and distribute rewards (e.g., rankings, perfect attendance award)
Personalized adjustments	Adjustment frequency	Assessed once a week (based on participation, medical condition, psychological state); temporary adjustments can be made in special circumstances (birthdays, low mood)
	Adjustment methods	1. Difficulty Adjustment: increase game difficulty (speed up the pace) after 3 consecutive sessions with ≥90% goal achievement; decrease difficulty (reduce tasks) after 2 consecutive sessions with ≤ 60% goal achievement 2. Content Adjustment: communicate with the child for 2–3 min daily to understand preferences and retain popular games

In the control group, health education focused on disseminating conventional rehabilitation knowledge. A 60-min centralized training session was also conducted at the start of the intervention. This session covered essential daily care points for recurrent pneumonia, such as maintaining warmth, dietary restrictions, and medication standards, alongside demonstrations of standard respiratory training movements like diaphragmatic and pursed-lip breathing. Nursing staff provided on-site guidance to family members, for instance, by showing them how to place hands gently on the child's abdomen to feel diaphragmatic movement and assess technique. During the intervention, a “regular guidance plus daily monitoring” format was implemented through targeted 15-min sessions held twice weekly. The first weekly session reviewed the completion of respiratory exercises and corrected any non-standard assistance from family members. The second session addressed the observation of the child's symptoms at home, explaining how to evaluate clinical changes through cough frequency and sputum characteristics, as well as appropriate responses to any abnormalities.

### Outcome measures

Pulmonary function was designated as the primary outcome measure, whereas exercise capacity and rehabilitation compliance were evaluated as secondary outcome measures.

#### Pulmonary function test

Test was performed before and after the intervention to measure forced vital capacity (FVC) and forced expiratory volume in one second (FEV1). The FEV1/FVC ratio was calculated, with a higher value indicating better lung function. Pulmonary function tests (PFTs) were performed in the standing position, in strict compliance with the Chinese guidelines for pulmonary function testing (11).

#### Exercise capacity

Exercise capacity was assessed using the 6-min walk test (6MWT). Children were instructed to walk back and forth as quickly as possible along a 30-meter, flat, straight corridor for 6 min. Standardized time reminders and encouragement were provided at regular intervals, and the total distance walked (6-min walking distance, 6MWD) was recorded.

#### Rehabilitation compliance and completion

Rehabilitation compliance was evaluated using the complementary Frankl and Houpt behavior rating scales (12). The Frankl scale rates a child's subjective willingness to cooperate on a 4-point scale from 1 (definitely negative) to 4 (definitely positive). The Houpt scale rates the actual effectiveness of treatment execution on a 6-point scale from 1 (treatment interrupted) to 6 (excellent).

### Data analysis

Two researchers independently entered the data using EpiData 3.1, and statistical analyses were performed using IBM SPSS 26.0. The statistician remained blinded to group allocation. Continuous variables were tested for normality using the Kolmogorov–Smirnov test. Normally distributed data are presented as mean ± standard deviation, whereas non-normally distributed data are presented as median and interquartile range. Homogeneity of variance was assessed using Bartlett's test. For normally distributed continuous variables, between-group comparisons were performed using independent-samples *t*-tests, and within-group comparisons between baseline and post-intervention values were performed using paired-samples *t*-tests. Non-normally distributed continuous variables were compared using the Mann–Whitney U test for between-group comparisons and the Wilcoxon signed-rank test for within-group comparisons. Categorical variables are presented as frequency and percentage and were compared using the chi-square test or Fisher's exact test, as appropriate.

## Results

### Completion of outcome assessments

**Figure 1:**
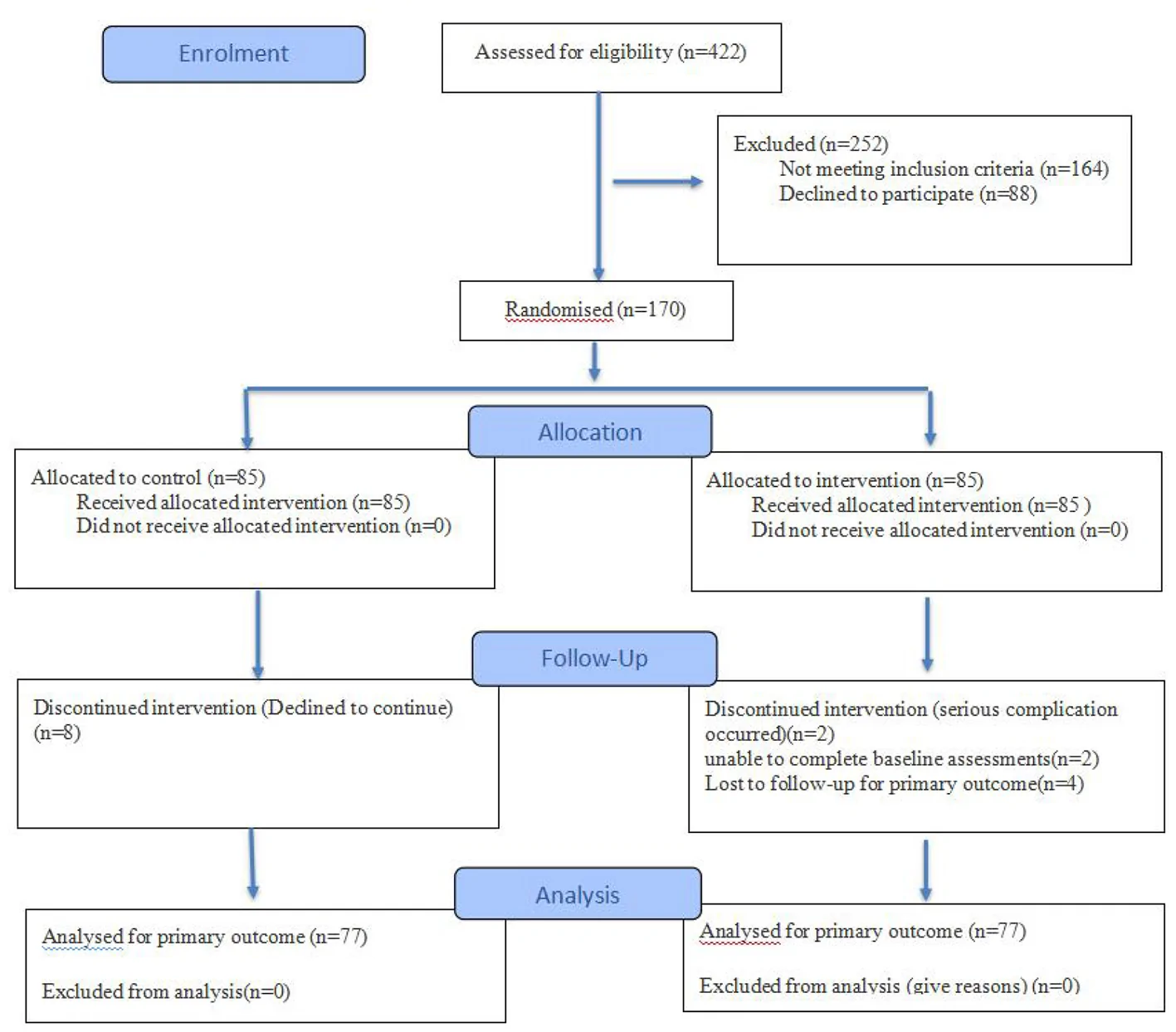
CONSORT flow diagram of participant enrollment.

As shown in the CONSORT flow diagram (Figure 1), 170 children were randomized after eligibility assessment. Among them, 2 children were unable to complete the baseline assessments and were therefore excluded from the final analysis. The reasons for incomplete baseline assessments may have included limited cooperation due to young age, difficulty understanding and following standardized assessment instructions, inability to perform technically acceptable respiratory maneuvers during pulmonary function testing, and temporary discomfort or fatigue during exercise assessment after pneumonia recovery. In some cases, children were unable to complete repeated attempts required to obtain reliable measurements because of limited attention span or anxiety associated with unfamiliar testing procedures.

All participants included in the final analysis completed the scheduled pulmonary function assessment and 6-min walk test at baseline and post-intervention.

### Baseline characteristics

No statistically significant differences were found in any baseline demographic or clinical characteristics between the groups (*P* > 0.05). The observation group was 54.5% male and 45.5% female, compared to 51.9% male and 48.1% female in the control group. Mean age was 6.8 ± 2.3 years in the observation group and 7.1 ± 2.1 years in the control group. Mean weight was 22.3 ± 5.8 kg and 21.6 ± 5.5 kg, respectively. Mean illness duration was 3.2 ± 1.5 days and 3.0 ± 1.4 days. Urban residents comprised 62.3% and 58.4% of the groups, respectively. Most guardians had a college education or higher (54.5% and 53.2%), with balanced distributions across lower education levels. The primary medical insurance for both groups was urban-rural resident insurance (>84%). The incidence of underlying diseases was 15.6% and 13.0%, respectively. This demographic consistency confirms the groups' comparability for the intervention study (Table 3).

**Table 3:** General participant characteristics (*N* = 154).

Characteristic	Intervention group (*n* = 77)	Control group (*n* = 77)	Statistic	*p*
Gender			χ^2^ = 0.153	0.695
Male	42 (54.5%)	40 (51.9%)		
Age	6.8 ± 2.3	7.1 ± 2.1	*t =* 0.785	0.434
Weight (Kg)	22.3 ± 5.8	21.6 ± 5.5	*t =* 0.912	0.363
Disease duration (days)	3.2 ± 1.5	3.0 ± 1.4	*t =* 0.821	0.413
Place of residence			χ^2^ = 0.387	0.533
City	48 (62.3%)	45 (58.4%)		
Rural	29 (37.7%)	32 (41.6%)		
Guardian's educational attainment			χ^2^ = 0.512	0.916
Primary school or below	5 (6.5%)	6 (7.8%)		
Junior high school	12 (15.6%)	11 (14.3%)		
High school/secondary vocational school	18 (23.4%)	19 (24.7%)		
Bachelor's degree or above	42 (54.5%)	41 (53.2%)		
Pre-existing condition			χ^2^ = 0.258	0.612
Yes	12 (15.6%)	10 (13.0%)		
No	65 (84.4%)	67 (87.0%)		

#### Pulmonary function

Pulmonary function was assessed using spirometry at baseline and after the intervention. Forced expiratory volume in 1 sec (FEV1) and forced vital capacity (FVC) were recorded as measured values in liters and were used to evaluate changes in pulmonary function over the study period (Table 4).

**Table 4:** Pre-post intervention outcomes ([[*Inline Image*]]±*s*).

	Fun games intervention			Usual care			Fun games intervention vs. usual care	
	Mean (SD)		Within-group change, mean (95% CI)	Mean (SD)		Within-group change, mean (95% CI)	Between-group difference, mean (95% CI)	*P* value
Outcome	Baseline	Post-intervention		Baseline	Post-intervention			
Primary outcome								
Pulmonary function	71.57 (3.14)	84.69 (2.34)	13.12 (12.89 to 13.33)	71.49 (2.93)	74.43 (2.27)	−2.94 (−3.11 to −2.75)	10.26 (9.53 to 10.99)	< 0.001
Secondary outcomes								
Exercise capacity	551.87 (25.25)	593.63 (19.91)	41.76 (34.43 to 49.08)	556.00 (31.49)	556.35 (30.48)	−0.34 (−10.21 to 9.52)	37.29 (29.09 to 45.48)	< 0.001
Frank scores	2.74 (0.55)	3.87 (0.34)	1.13 (1.02, 1.24)	2.79 (0.50)	3.10 (0.60)	−0.31 (−0.41 to 0.21)	0.77 (0.61 to 0.92)	< 0.001
Houpt scores	3.27 (1.68)	3.85 (1.45)	0.58 (0.13 to 1.04)	3.46 (1.71)	2.58 (1.05)	0.88 (0.44 to 1.32)	1.27 (0.87 to 1.67)	< 0.001

#### Exercise capacity

Exercise capacity was measured via the 6-min walking distance (6MWD). Each child walked as quickly as possible along a 30-meter straight, flat course for 6 min. Standardized verbal encouragement and time reminders were provided throughout the test. The final position was recorded as the endpoint (Table 4).

#### Compliance and completion

Compliance was evaluated using both the Frankl and Houpt behavior rating scales, which assess subjective willingness and actual procedural completion, respectively. The Frankl scale rates cooperative willingness on a 4-point scale: 1 (definitely negative), 2 (negative), 3 (positive), and 4 (definitely positive). The Houpt scale rates the behavioral impact on procedure execution on a 5-point scale from 1 (excellent, treatment completed) to 5 (aborted, no treatment rendered). The Houpt scale is a behavioral rating tool used to assess a child's cooperation during treatment, with higher scores indicating better behavior and more successful procedure completion (Table 4).

There were no statistically significant differences in all baseline variables between the groups (*P* > 0.05).

## Discussion

In this single-center randomized controlled trial, a game-based intervention significantly improved both adherence to respiratory rehabilitation and clinical outcomes in children recovering from pneumonia compared with conventional respiratory rehabilitation. The 8-week intervention led to marked enhancements in respiratory function, treatment adherence, and completion rates. This approach is straightforward to implement, economically efficient, and demonstrably effective, supporting its broader clinical application to improve the care experience and therapeutic results for this patient population. These findings confirm our initial hypothesis and are consistent with the beneficial effects of play therapy documented in children with autism spectrum disorder (13).

FEV1/FVC is a commonly used spirometric index reflecting expiratory airflow relative to forced vital capacity. In this study, spirometry was selected as the primary pulmonary outcome because it is a standardized, non-invasive, and clinically feasible method for evaluating respiratory function recovery in children who can perform reproducible respiratory maneuvers. Compared with more advanced pulmonary function assessments, such as impulse oscillometry, body plethysmography, or diffusion capacity testing, spirometry requires relatively simple equipment, lower operational costs, and is more widely applicable in routine pediatric rehabilitation settings. Although both groups showed increased FEV1/FVC values after the intervention, the improvement was greater in the intervention group. This result suggests a potential beneficial effect of the game-based intervention on pulmonary function. Nevertheless, FEV1/FVC alone does not fully capture pneumonia severity or recovery, and the clinical relevance of the observed change should therefore be interpreted cautiously. To further substantiate these findings, we incorporated the 6-min walk test (6MWT), a validated and sensitive measure of functional status. Given that pulmonary inflammation is often associated with elevated D-dimer levels and can compromise exercise tolerance, we also incorporated the 6-min walk test (6MWT) as an evaluative measure. The 6MWT has been established as a sensitive and comprehensive tool for assessing functional status. On this indicator as well, the experimental group showed significant improvement in this study (14). This correlation suggests the intervention may promote comprehensive recovery of a child's activity capacity by mitigating inflammation and optimizing gas exchange, beyond its local effects on respiratory function.

Our study further underscores the critical importance of treatment adherence and completion for patient recovery. Play therapy facilitates emotional expression and promotes psychological stabilization, which corroborates the conclusions of several meta-analyses (15, 16). This result, however, contrasts with another study that found no significant effect of a game intervention on adherence (4). In contrast, the game-guided approach was associated with higher adherence and completion rates in the present study.

Some studies note that while standardized treatment and chest physiotherapy help reduce pneumonia mortality, they do not significantly shorten hospital length of stay (17). Consequently, our design incorporated family involvement and explanations of rehabilitation principles, which likely helped children cooperate with and complete the overall treatment plan more effectively.

The results of this study hold important significance within the clinical context of pediatric pneumonia rehabilitation. By integrating a multidisciplinary framework from psychology, behavioral science, and rehabilitation medicine, we have outlined a coherent intervention pathway. Future research should build upon this foundation to further improve rehabilitation quality. The family, as a fundamental unit in Chinese society, has shown positive effects in family-centered intervention models across various patient populations (18, 19). Therefore, while fully considering family cultural backgrounds, play therapy could be integrated with internet platforms to deliver online rehabilitation guidance. Online interventions have shown good feasibility and acceptability, with the potential to conserve therapist time and reduce healthcare costs while expanding access for children in resource-limited areas, although their efficacy requires further validation.

Although this study achieved its objectives, several limitations must be acknowledged. This study has several limitations. First, its single-center design and relatively homogeneous sample of 154 patients may introduce selection bias and limit generalizability. Second, the intervention period was short, with outcomes assessed only up to 8 weeks; therefore, long-term efficacy and delayed adverse events remain unclear. Third, data were collected only at baseline and after intervention, preventing analysis of temporal trends. Fourth, adherence was assessed mainly using subjective scales, which may be affected by reporting bias. Fifth, participants could not be blinded because of the nature of the intervention, which may have introduced performance bias. Finally, the intervention protocol was not stratified by age group, limiting its applicability across developmental stages. In addition, FEV1/FVC were reported as measured values rather than as percent predicted values or z-scores, and spirometry itself has limitations in younger children with limited cooperation, with FEV1/FVC alone unable to fully capture all dimensions of pulmonary recovery after pneumonia. Future multicenter studies should include longer follow-up, multiple assessment time points, objective adherence monitoring, age-stratified protocols, blinded outcome assessment, standardized pediatric spirometry reporting based on prospectively collected anthropometric data, and ideally combine multiple pulmonary function tests to provide a more comprehensive evaluation.

This study demonstrates the feasibility and effectiveness of this intervention within the clinical and cultural context. These findings are expected to provide new insights for the clinical nursing practice of pediatric pneumonia and offer a theoretical reference for delivering more humanistic rehabilitation services.

## Data Availability

The raw data supporting the conclusions of this article will be made available by the authors, without undue reservation.
